# Poster Session II - A204 GASTRIC INLET PATCH PREVALENCE & ITS ASSOCIATION WITH GLOBUS SENSATION: ROLE OF INTENTIONAL ESOPHAGEAL INSPECTION

**DOI:** 10.1093/jcag/gwaf042.204

**Published:** 2026-02-13

**Authors:** C Liu, A Matias, L Hookey

**Affiliations:** Queen’s University, Kingston, ON, Canada; University of Toronto, Toronto, ON, Canada; Queen’s University, Kingston, ON, Canada

## Abstract

**Background:**

Globus sensation is a persistent, non-painful feeling of a lump in the throat with an unclear etiology. One proposed cause is the gastric inlet patch (GIP), a heterotopic gastric mucosa found in the cervical esophagus. Although typically asymptomatic, emerging literature suggests a potential association between GIP and globus sensation, particularly in symptomatic cases that secrete acid similarly to gastroesophageal reflux disease (GERD). However, the prevalence of GIP varies significantly across studies, partly due to inconsistent reporting by endoscopists and a lack of standardized detection protocols.

**Aims:**

To determine the prevalence of GIP and compare detection rates between retrospective endoscopies performed without intentional investigation and prospective endoscopies in which endoscopists were instructed to actively inspect for GIP, and explore the relationship between GIP and gastrointestinal symptoms.

**Methods:**

Upper endoscopy reports from patients at Kingston Health Sciences Centre were analyzed. The retrospective cohort included procedures performed in 2024 without specific instruction to assess for GIP. The prospective cohort included endoscopies where physicians intentionally inspected the proximal esophagus for GIP. Cohort differences were evaluated using Fisher’s exact test. Patient demographics, upper gastrointestinal symptoms, and antacid use were recorded.

**Results:**

In the retrospective cohort, 161 patient records were analyzed, revealing a GIP prevalence of 1.24% (95% CI: 0.15%-4.42%). In the prospective cohort, 150 patients were intentionally investigated, revealing a GIP prevalence of 2.67% (95% CI: 0.73%-6.78%). There was no statistical difference (P = 0.68) between retrospective and prospective cohorts. Among the seven study patients with GIP, complete symptom data wwas available for six. All six reported dysphagia, and three (50%) reported gastric reflux.

**Conclusions:**

Intentional inspection of the proximal esophagus did not significantly increase GIP detection, confirming a low true prevalence in our population. This study provides a regional comparison of standard versus intentional endoscopy for GIP detection. Among identified cases, patients reported dysphagia, highlighting the need for further research to clarify the clinical relevance of GIP and its relationship with upper gastrointestinal symptoms.

**Funding Agencies:**

None

